# Dosiomics and radiomics to predict pneumonitis after thoracic stereotactic body radiotherapy and immune checkpoint inhibition

**DOI:** 10.3389/fonc.2023.1124592

**Published:** 2023-03-15

**Authors:** Kim Melanie Kraus, Maksym Oreshko, Denise Bernhardt, Stephanie Elisabeth Combs, Jan Caspar Peeken

**Affiliations:** ^1^ Department of Radiation Oncology, School of Medicine and Klinikum rechts der Isar, Technical University of Munich (TUM), Munich, Germany; ^2^ Institute of Radiation Medicine (IRM), Helmholtz Zentrum München (HMGU) GmbH German Research Center for Environmental Health, Neuherberg, Germany; ^3^ Partner Site Munich, German Consortium for Translational Cancer Research (DKTK), Munich, Germany; ^4^ Medical Faculty, University hospital, Ludwig-Maximilians-Universität (LMU) Munich, Munich, Germany

**Keywords:** pneumonitis, SBRT (stereotactic body radiation therapy), radiomics, dosiomics, immune checkpoint inhibition, model based prediction, lung cancer

## Abstract

**Introduction:**

Pneumonitis is a relevant side effect after radiotherapy (RT) and immunotherapy with checkpoint inhibitors (ICIs). Since the effect is radiation dose dependent, the risk increases for high fractional doses as applied for stereotactic body radiation therapy (SBRT) and might even be enhanced for the combination of SBRT with ICI therapy. Hence, patient individual pre-treatment prediction of post-treatment pneumonitis (PTP) might be able to support clinical decision making. Dosimetric factors, however, use limited information and, thus, cannot exploit the full potential of pneumonitis prediction.

**Methods:**

We investigated dosiomics and radiomics model based approaches for PTP prediction after thoracic SBRT with and without ICI therapy. To overcome potential influences of different fractionation schemes, we converted physical doses to 2 Gy equivalent doses (EQD2) and compared both results. In total, four single feature models (dosiomics, radiomics, dosimetric, clinical factors) were tested and five combinations of those (dosimetric+clinical factors, dosiomics+radiomics, dosiomics+dosimetric+clinical factors, radiomics+dosimetric+clinical factors, radiomics+dosiomics+dosimetric+clinical factors). After feature extraction, a feature reduction was performed using pearson intercorrelation coefficient and the Boruta algorithm within 1000-fold bootstrapping runs. Four different machine learning models and the combination of those were trained and tested within 100 iterations of 5-fold nested cross validation.

**Results:**

Results were analysed using the area under the receiver operating characteristic curve (AUC). We found the combination of dosiomics and radiomics features to outperform all other models with AUC_radiomics+dosiomics, D_ = 0.79 (95% confidence interval 0.78-0.80) and AUC_radiomics+dosiomics, EQD2_ = 0.77 (0.76-0.78) for physical dose and EQD2, respectively. ICI therapy did not impact the prediction result (AUC ≤ 0.5). Clinical and dosimetric features for the total lung did not improve the prediction outcome.

**Conclusion:**

Our results suggest that combined dosiomics and radiomics analysis can improve PTP prediction in patients treated with lung SBRT. We conclude that pre-treatment prediction could support clinical decision making on an individual patient basis with or without ICI therapy.

## 1 Introduction

High precision stereotactic body radiation therapy (SBRT) is common standard for treatment of early stage inoperable lung cancer as well as for pulmonary oligo-metastases with excellent local control and an acceptable toxicity profile (1–4). While immunotherapy including checkpoint inhibitors (ICIs) substantially improved the outcome for early lung cancer patients with regard to local tumor control and overall survival (5), the impact of combination with thoracic radiotherapy remains unclear with regard to the development of side effects. PTP is a rather frequent and dose limiting side effect of both, radiation and ICI therapy. As the development of PTP is dose dependent, the risk increases for high fractional doses as applied by SBRT (6). In contrast to the majority of data in the literature, there is also evidence of increased all grade pneumonitis rates (5, 7, 8) after combined radioimmunotherapy with ICIs. This might be of relevance for decision making with regard to further therapeutic options on a patient individual basis.

The applied radiation dose is the most important factor for radiation-dependent pneumonitis. Dose volume histograms (DVHs), however, cannot account for the spatial distribution of the dose and potential effects on the tissue. Thus, prediction of the risk for the development of PTP relying on the spatial distribution could gain clinical advantage for individual patient treatment. Apart from conventional dosimetric approaches, sophisticated methods such as machine learning gain more and more importance for radiation oncology. In recent years, it has been shown that spatial quantitative features assessing the image grey-level distribution extracted from medical imaging data (radiomics) allow for unprecedented predictions of clinical endpoints including patient survival, disease progression, tumor characterization, tumor response and tumor detection (9–17). Analysis using spatial features of the dose distribution or image grey-level distributions, referred to as dosiomics (18–22) or radiomics (23–25) and even the combination of both (26, 27) have also been successfully investigated for prediction of lung toxicity after thoracic radiotherapy in previous studies.

The radiomics features based on pretreatment computed tomography (CT) data showed improvement to predict high grade radiation pneumonitis after definitive radiotherapy (23, 25) and after SBRT (24). Several studies investigated lung toxicity prediction for normofractionated radio(chemo)therapy (RCT). Liang et al. compared dosiomics prediction of radiation pneumonitis after primary thoracic radiotherapy with dosimetric and normal tissue control possibility (NTCP) models and found dosiomics to surpass all other methods (20). In a similar approach, Bourbonne et al. also found dosiomics models to outperform clinical and dosimetric models for prediction of lung toxicity (18). Additionally, combination of radiomics and dosiomics models could even improve the prediction of radiation pneumonitis (26) and for SBRT, other studies support these findings. Jiang et al., additionally revealed improved prediction by machine learning models using dosiomics for different anatomical regions of interest (27), however only for normofractionated radiation schemes. Adachi et al. also tested dosiomics against dosimetric models and against a hybrid model of both resulting in best prediction of radiation pneumonitis achieved with the dosiomics model (19).

These studies investigated PTP prediction after normofractionated R(C)T or SBRT using radiomics and dosiomics combined or dosiomics, respectively. In addition to the above summarized findings, with this study, we aim to find the potential value for the occurrence of PTP after thoracic SBRT using the combination of radiomics and dosiomics analysis of 3D dose distributions and CT data. Additionally, we investigate the potential impact of combined radioimmunotherapy with ICIs.

## 2 Methods

### 2.1 Clinical factors

**Figure 1:**
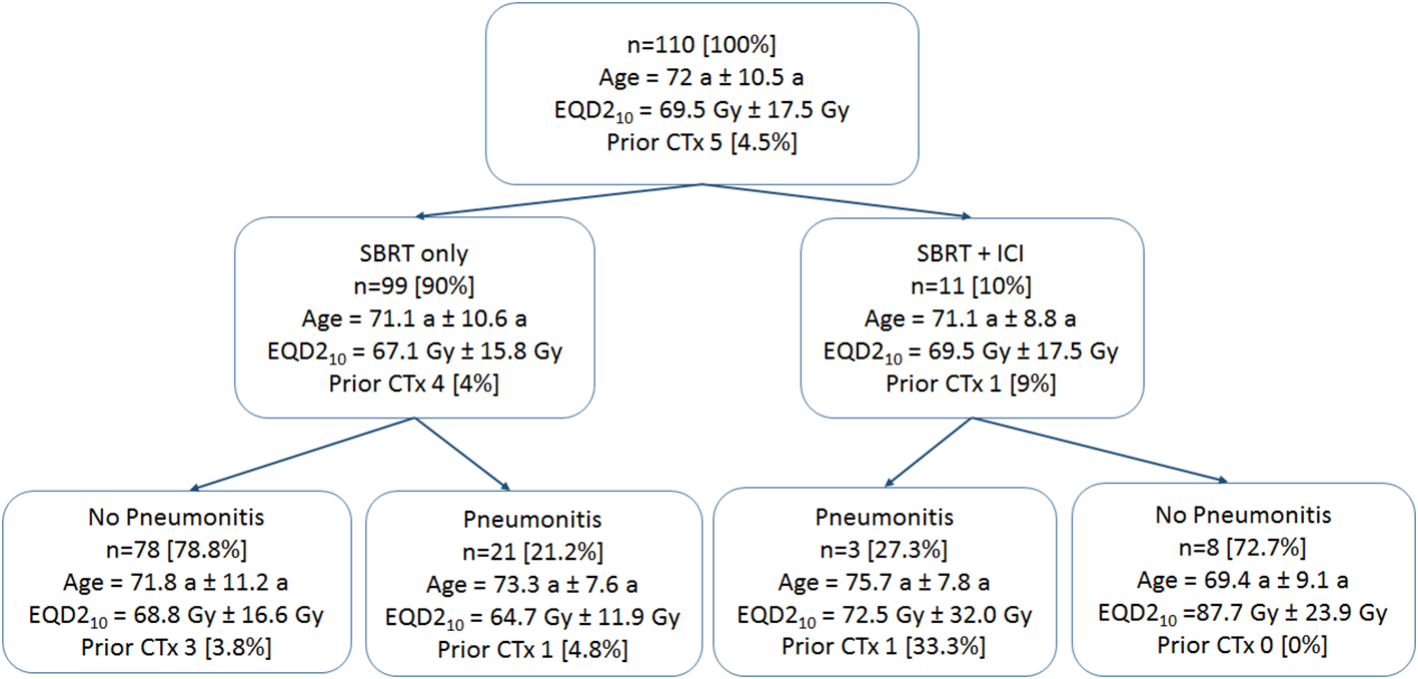
Patient data groups. Patient mean age and standard deviations are provided. Prescription doses are given in mean values and standard deviations of equivalent uniform doses for an α/β of 10 Gy (EQD2_10_). The number of patients who received prior chemotherapy (CTx) is provided.

A total of 110 cases of primary lung cancer or pulmonary metastases received SBRT between 2010 and 2021. All patients provided written informed consent before enrollment. Dose and fractionation schemes varied with fraction doses ranging between 5 Gy and 15 Gy. Patient data involving patient age, sex, karnofsky performance index (KPI), tumor location and size, previous chemotherapy and ICI therapy within 50 days around SBRT. The occurrence of post-treatment pneumonitis (PTP) of all grades according to the Common Terminology Criteria for Adverse Events version 5.0 (28) was detected in follow-up CT scans and from corresponding clinical findings (e.g. dyspnea, cough, pain) during follow-up visits monitored in the patient files. An overview of the patient data is provided in **Figure 1**.

### 2.2 CT and dose data

Radiotherapy planning CTs, 3D dose distributions, lung and treatment volume segmentations as well as dose volume histogram (DVH) data were selected from the radiotherapy treatment planning system Eclipse (Varian, Paolo Alto). Patients received a 4D-CT prior to radiotherapy. A gross tumor volume (GTV) was delineated on ten phase CTs. Subsequently, an internal target volume was generated which encompasses the GTV across all ten 4D-CT phases. An additional margin of up to 5 mm was added to the internal target volume resulting in the planning target volume (PTV).

Dosimetric data for the total lung included mean dose, the volume receiving at least 5 Gy (V5) and V10, V15, V20, V30, V40, V50, accordingly (29). Required post processing of the segmentation data was performed using the open source platform 3D Slicer (30) and the Radiation Therapy toolkit (31). To take the impact of different fractionation schemes into account, physical dose distributions as extracted from Eclipse were converted into 2 Gy fractions equivalent doses (EQD2) on a voxel basis using an in-house developed Matlab tool (32) according to equation (1) where *D* is the sum dose over all fractions, *d* is the fraction dose, and $\frac{\alpha }{\beta }$ is equal to 3 for lung tissue. Dose outside the lung was not considered.


(1) $$EQD2=D\left[\frac{d+\frac{\alpha }{\beta }}{2+\frac{\alpha }{\beta }}\right]$$

### 2.3 Feature extraction

From each volume of interest (total lung minus GTV, ipsilateral lung minus GTV, PTV + 2cm isotropic margin) 104 radiomics and dosiomics features were extracted from the planning CT and 3D dose distributions using the open-source library Pyradiomics in Python (see **Supplemental Table 1** for a list of all features) leading to 312 features, respectively (33). 3D dose maps were treated as images with Gy values as grey-levels. Feature reduction was performed within 1000-fold bootstrapping using pearson intercorrelation coefficient with a cut-off value of 0.7 (arbitrarily chosen to allow sufficient input features for all feature sets) and the Boruta algorithm as previously described (34). In brief, the Boruta algorithm iteratively removes features that appear unimportant for the prediction of the PTP in comparison to synthetic random features (35). The features were ranked according to the frequency of selection overall bootstrap runs. The final feature set was defined as the top-ranking features. The final feature number per model was defined as the median feature number selected over all bootstrap runs. For combined models, the preselected features from each group were used as input for the same procedure.

### 2.4 Machine learning models

**Figure 2:**
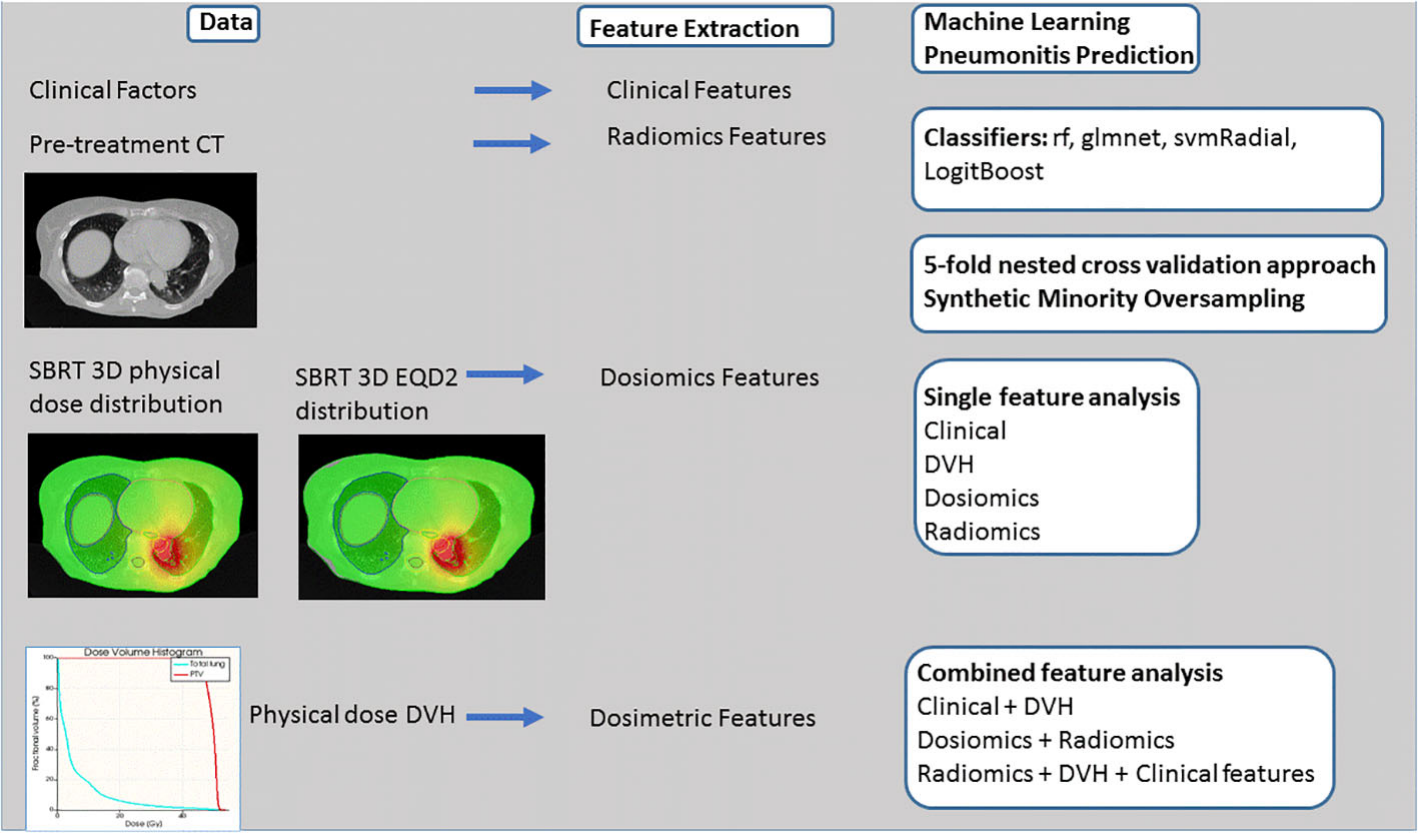
Process flow. Clinical, Computed Tomography (CT) and 3D dose volume and dose volume histogram (DVH) data is used for feature extraction. PTP prediction is performed testing different classifiers such as random forest (rf), logistic elastic net regression (glmnet), support vector machine (svmRadial), and logitBoost and 5-fold nested cross validation approach and Synthetic Minority Oversampling. Four single models and five combined models are analyzed.

The entire process flow is depicted in **Figure 2**. Three single predictive models (radiomics, dosiomics, clinical factors) and five combined models (dosiomics + radiomics, DVH + clinical factors, radiomics + DVH + clinical data, dosiomics + DVH + clinical data, all) were investigated for the physical dose and EQD2 dose distributions. Different machine learning models with in-built feature reduction including random forest (rf), logistic elastic net regression (glmnet), support vector machine (svmRadial), and logitBoost were trained and tested using 100 iterations of 5-fold nested cross validation in R according to Deist et al. (36). This led to training/test splits of 88:22 and 70:18 in the outer and inner folds, respectively. Due to class imbalance, Synthetic Minority Oversampling Technique (SMOTE) resampling was applied based on the R DMwR package (37) introducing data augmentation of the minority class *via* generation of synthetic samples using a k-nearest neighbor approach and undersampling of the majority class. Due to the small event number, a k-value of 3 was chosen for the k-nearest neighbor procedure. The ratio of oversampling and undersampling was empirically optimized leading to “perc.over” and “perc.under” equaling to the default value of 200%. For comparison, all machine learning models were also calculated without any weighting or SMOTE resampling (see **Supplemental Table 3**). Hyperparameter optimization was performed within the inner folds using grid search (see **Supplemental Table 4** for Hyperparameter Space). Single feature models (e.g. ICI) were modeled using logistic regression. The entire process flow is depicted in **Figure 2**. Model performance was analysed using the area under the receiver operating characteristic curve (AUC) on the test sets of the outer folds. Data is presented as mean values and confidence intervals with a confidence level of 95%. For comparison of different classifiers used, AUC values were calculated for each dataset and repetition and were ranked by ordering between numbers ranging from 1 to 4 for the four different single predictive models. Data is presented in box and scatterplots as ranked AUC values with each point representing the result of one outer validation fold.

## 3 Results

### 3.1 Comparison of classifiers

**Figure 3:**
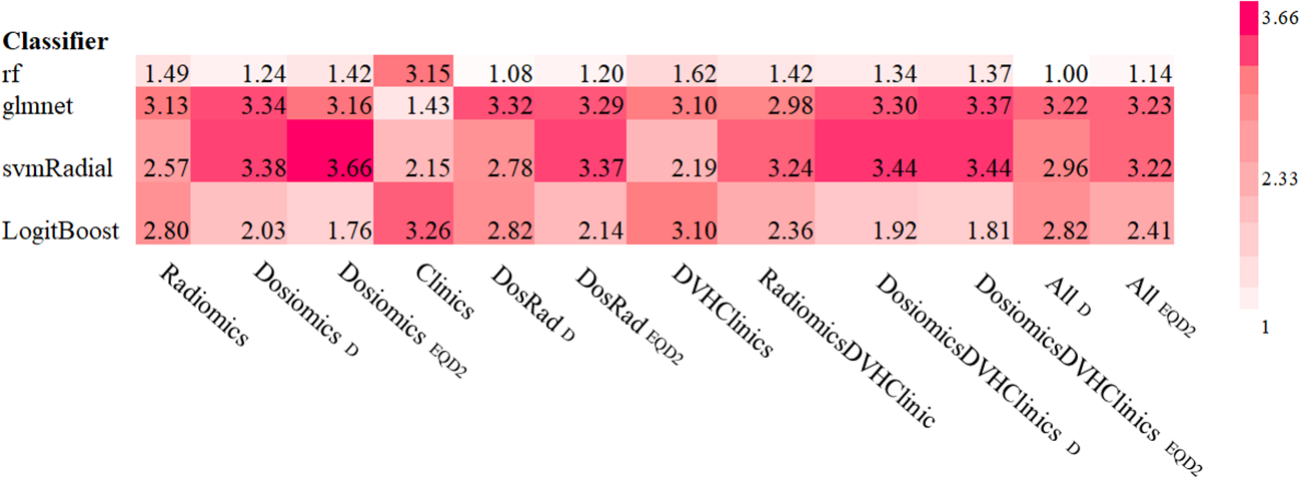
Ranked mean AUC values for all classifiers and models tested. Subscripted D and EQD2 refer to physical dose and EQD2, respectively.

Comparison of different classifiers revealed rf to perform best for all models tested resulting in a mean AUC rank value of 1.08 and 1.20 for physical dose and EQD2 analysis. **Figure 3** shows the ranked AUC values for all applied classifiers. Based on these findings, for the following analyzes, we chose rf.

### 3.2 Clinical factors

A summary of the clinical parameters collected and the patient groups is given in **Table 1** and **Figure 1**. Most tumors occurred in the right upper lung (30 (27.3%)). A total of 10% of patients received additional ICI therapy. Five patients received primary lung cancer treatment, however, all in a metastasized stage, and six were treated due to metastases. Most of the patients (95%) did not receive previous chemotherapy. Pneumonitis occurred in 24 (21.8%) of all patients, 12.5% (3) of them received additional ICI therapy and 87.5% (21) did not receive additional ICI therapy.

**Table 1:** Clinical factors.

Characteristic	Value	Value [%]
Age		
Mean ± SD	72 ± 10.48	
Range	33-90	
Sex		
Male	69	62.7
Female	41	37.3
KPI		
Mean ± SD	95 ± 5.90	
Range	80-100	
Tumor size		
Mean ± SD	61162.1 cc ± 75582 cc	
Range	4601.3 cc-524554 cc	
Location		
RUL	30	27.3
RML	2	1.8
RLL	25	22.7
LUL	36	32.7
LLL	11	10.0
RC	3	2.7
LC	3	2.7
SBRT+ICI		
Yes	11	10.0
No	99	90.0
Prior CTx		
Yes	5	4.5
No	105	95.5
Pneumonitis		
Yes	24	21.8
No	85	77.3

### 3.3 Feature extraction

All features used for feature extraction are listed in **Supplement Table 1**. The reduced extracted features for all models tested are provided in **Supplement Table 2**. There was no correlation between ICI and the selected features within the model combining all features. In total, four clinical features were extracted and ranked as follows: tumor size, patient age, tumor location and patient sex. From dosimetric parameters, only V50 and V5 were selected for physical dose and EQD2 features, respectively. Combining both models resulted just in the combination of all single feature models.

Across all model analyzes, 17 to 33 features were found. The most relevant features are listed in **Table 2**.

**Table 2:** Features ranked in the order of frequency they have been selected after feature reduction for all models tested.

Model	Number of reduced features	Ranked features
Radiomics	21	PTV_original_shape_Sphericity
		Total_Lung_original_glcm_Idn
		Ispilateral_Lung_original_glcm_InverseVariance
Dosiomics_D_	17	PTV_original_shape_Sphericity
		Total_Lung_original_shape_Flatness
		PTV_original_glcm_Idmn
Dosiomics_EQD2_	17	PTV_original_shape_Sphericity
		Total_Lung_original_shape_Flatness
		PTV_original_glcm_Idmn
Radiomics + Dosiomics_D_	28	PTV_original_shape_Sphericity
		PTV_original_glszm_SmallAreaLowGrayLevelEmphasis
		Ipsilateral_Lung_original_glcm_InverseVariance
Radiomics + Dosiomics_EQD2_	28	PTV_original_shape_Sphericity
		PTV_original_glcm_Idmn
		Ispilateral_Lung_original_glcm_InverseVariance
Radiomics + Clinical Factors + DVH	27	PTV_original_shape_Sphericity
		Total_Lung_original_glcm_Idn
		Ispilateral_Lung_original_glcm_InverseVariance
Dosiomics_D_ + Clinical factors + DVH	22	PTV_original_shape_Sphericity
		Total_Lung_original_shape_Flatness
		Total_Lung_original_shape_Elongation
Dosiomics_EQD2_ + Clinical factors + DVH	22	PTV_original_shape_Sphericity
		Total_Lung_original_shape_Flatness
		Total_Lung_original_shape_Elongation
Radiomics +Dosiomics_D_ + Clinical factors + DVH	33	PTV_original_shape_Sphericity
		PTV_original_glszm_SmallAreaLowGrayLevelEmphasis
		Ispilateral_Lung_original_glcm_InverseVariance
Radiomics +Dosiomics_DEQD2_ + Clinical factors + DVH	33	PTV_original_shape_Sphericity
		PTV_original_glcm_Idmn
		Ispilateral_Lung_original_glcm_InverseVariance

Subscripted EQD2 refers to the equivalent dose in 2 Gy fractions and D to the physical dose.

### 3.4 Prediction model performance

#### 3.4.1 Single feature models

**Figure 4:**
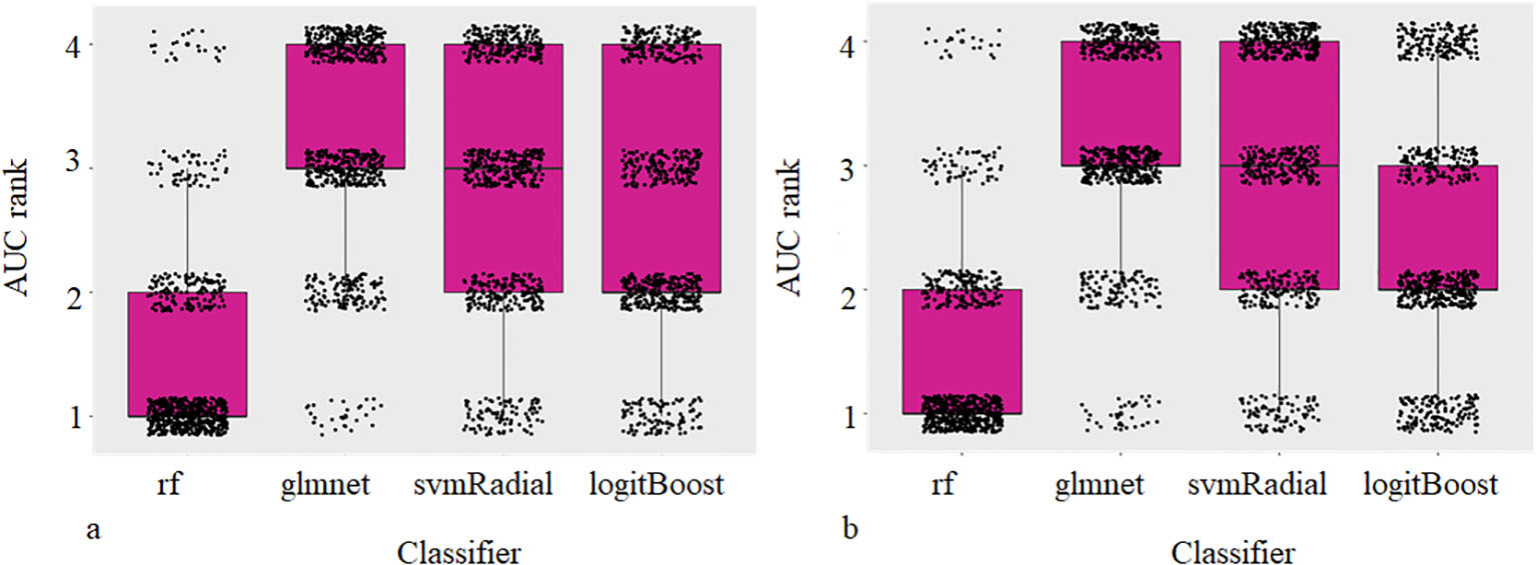
Box and Scatterplots showing area under the receiver operating characteristic curves (AUCs) rank values (lower being better) for different classifiers used over all datasets and repetitions for physical (a) and EQD2 dosiomics analysis (b).

For both, physical dose and EQD2, dosiomics models predicted PTP better than random with AUC_dosiomics, EQD2_ = 0.68 (0.67-0.70) and AUC_dosiomics,D_ = 0.70 (0.68-0.71), respectively. The radiomics model achieved the highest predictive value (AUC_radiomics,D_ = 0.73 (0.72-0.74)). Other classifiers resulted in worse predictive results depicted in **Figure 4**. DVH parameters achieved PTP prediction yielding no better than random (AUC = 0.43 (0.42-0.46)). Clinical data and ICI therapy status was not predictive for the development of PTP, independent from the applied classifier (AUC = 0.45 (0.44-0.47) and AUC = 0.46 (0.42-0.44)), respectively.

#### 3.4.2 Combined feature models

**Figure 5:**
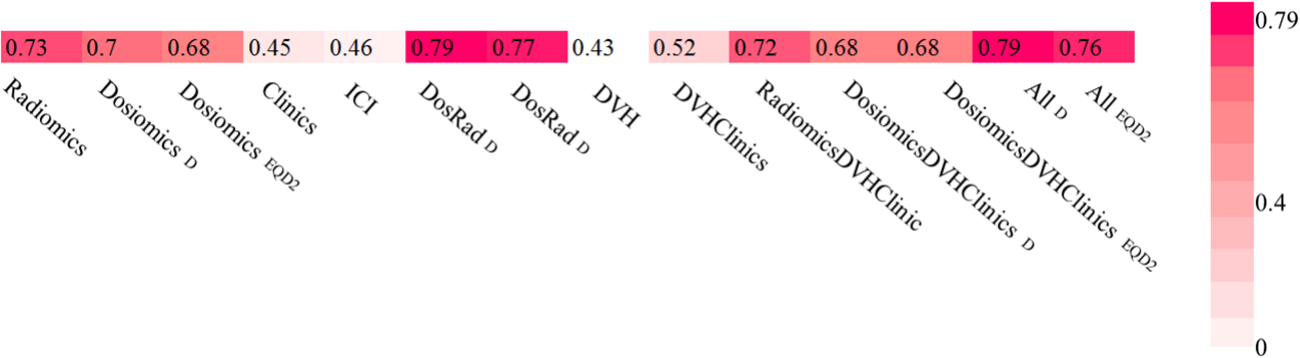
Area under the receiver operating characteristic curves (AUCs) heat maps as prediction substitute for PTP for physical Dose and EQD2 using random forest classifier and logistic regression for single feature models.

For the combination of radiomics and dosiomics, PTP was predicted better than random with AUC_radiomics+dosiomics, D_ = 0.79 (0.78-0.80) and AUC_radiomics+dosiomics, EQD2_ = 0.77 (0.76-0.78) for both, physical dose and EQD2, respectively. Combination with other models including ICI therapy and clinical data did not improve the prediction model. Results are depicted in **Figure 5**.

## 4 Discussion

Our results indicate that additional ICI therapy has no impact on the prediction of PTP after thoracic SBRT. PTP prediction can be improved by combining radiomics and dosiomics features. This combination outperformed radiomics-only and dosiomics-only models as well as DVH and clinical parameters and can improve prediction of PTP after thoracic SBRT.

In our work, the dosiomics feature model surpassed all clinical and DVH models with an AUC of 0.70 and 0.68 for physical dose and EQD2. These results are well in line with findings in the current literature. For example, in the study of Liang et al. dosiomics analysis with an AUC of 0.78 also resulted in favorable results when compared to dosimetric and NTCP factors (20). Importantly, in our study, prediction of PTP after thoracic SBRT could even be improved when dosiomics features were combined with radiomics features, which has not been previously shown for patients receiving lung SBRT. Two other works studying patients receiving lung RCT showed combined radiomics and dosiomics models to outperformed single feature class models with an AUC of 0.68 and 0.88 for radiomics and dosiomics combination models, respectively (26, 38). Jiang et al. found the combination of radiomics, dosimetrics, age and tumor T stage to result in a further increased AUC of 0.94.

The total performance of our model with a maximum AUC of 0.79 for the combined radiomics/dosiomics model is well in line with other studies on PTP prediction (20, 24, 26). A few studies, however, achieved larger predictive AUC values above 0.90. Several reasons may explain this fact: 1) The majority of other studies tested prediction of grade ≥ 2 pneumonitis, whereas we tested prediction of all grades of pneumonitis. The reason for this choice of data inclusion was triggered by unknown potential interfering effects associated with the combination of SBRT with immunotherapy that should not be overseen at this stage. Hence, we considered any detectable lung damage or symptom associated with pneumonitis worthwhile to include in our data set. 2) We applied a sophisticated nested cross validation approach separating the validation cohorts for hyperparameter optimization from the actual testing cohort. By iterating the process 100 times, statistical robustness was achieved. This procedure reduces the risk of overly optimistic results that may derive from small test sets or simple cross validation approaches (18, 19, 27).

The DVH features extracted were expected to be comparable with commonly known dosimetric risk factors for radiation pneumonitis such as mean lung dose, the lung volume receiving a dose of 10 Gy and 20 Gy, V10 and V20, respectively. Palma et al. found V20 to be predictive for grade ≥ 2 radiation pneumonitis after radiochemotherapy (39). Tsujino et al. found V20 and Fay et al. V30 and mean lung dose to be most predictive for symptomatic radiation pneumonitis after radiotherapy (40, 41). However, in our study only V50 and V5 were selected by feature extraction and did not predict PTP better than random (AUC< 0.5) in contrast to previous works (18, 19, 24). Different from other studies, we included all grades of pneumonitis into our analysis which could lead to differing dosimetric parameters or even missing correlation of common dosimetric parameters and the development of PTP. In our study, the highest grade of PTP observed was grade 2 in three patient cases and out of these one received additional ICI therapy. Due to the retrospective character of this investigation, the probability of misgrading increases. Our SBRT fractionation schemes cover a rather large range including single doses with a minimum of 5 Gy and lower total doses addressed to treat metastatic disease less likely to cause PTP.

Addition of clinical factors did not improve the prediction of pneumonitis. Likewise, Krafft et al. observed clinical characteristics to not improve the prediction model for high grade pneumonitis after definitive radiotherapy with conventional fractionation (23).

We converted doses to 2 Gy equivalent doses in order to compare different fractionation schemes applied and compared prediction outcome for dosiomics models based on physical dose and biological dosiomics features. As expected, results were comparable with a mean AUC of 0.7 and 0.68 for single dosiomics features analysis using physical dose and EQDs, respectively. This is well in line with findings in the literature (42). However, EQD2 could not further improve the prediction leading to the conclusion that conversion into EQD2 might be unnecessary for PTP prediction.

Development of machine learning models in a dataset of 110 patients is a challenging task, especially when considering the observed imbalance of the predicted outcome. To be able to test our medical hypothesis with regard to the comparison of the predictive values of different feature sets, we decided for several technical steps to allow for optimal training and testing the limitations and reduce the risk of overfitting: 1) we compared multiple machine learning algorithms to determine the algorithm best suited to learn from the small dataset; 2) we applied a cross validation approach with 5 folds to ensure a minimum of samples in the patients subgroups; 3) we applied SMOTE to decrease the influence of the imbalanced outcome variable; 4) we applied multiple feature reduction steps to reduce the feature space to the most predictive features per feature set; 5) no assumption of the optimal number of features was made beforehand; 6) we applied a nested-cross validation approach allowing for repeated testing on unseen data, completely independent of the data used for hyperparameter optimization. Finally, our models achieved good predictive performances in the range of multiple previous works as discussed above. Comparison of the results calculated without any weighting or SMOTE resampling did not change the presented result. Thus, the choice of data augmentation did not alter the relevant comparison of the analyzed models. Importantly, all prediction models were trained and tested simultaneously using the same technical principles and patient subsets down to the internal cross validation folds, guaranteeing optimal comparability. As consequence, the limitations of the model development were the same for all models – allowing for a fair comparison of the predictive value of the underlying feature sets.

Obvious limitations of this study are the retrospective character of data collection. Prospective data could improve the data quality with regard to PTP definition. Patients in this study receiving ICI therapy where all in a metastasized tumor stage. Clearly, this could lead to an imbalance between the SBRT only and the SBRT plus ICI group with slightly enhanced PTP rates (27.3% *vs*. 21.2%) in the combined therapy group. Additionally, there is a lack of patients included in the ICI group resulting a paucity of PTP events. Very few patients were diagnosed with pneumonitis grade ≥ 2, which could limit the clinical relevance of the prediction results. In our study, we decided to include all grade pneumonitis. One reason for this choice was to account for unknown effects occurring during combined radioimmunotherapy, and another reason was the uncertainty of grading coming along with retrospective data collection. Further, we did not apply external test data. External validation, however, is necessary to demonstrate reproducibility of models which is planned in future.

## 5 Conclusions

We demonstrated the potential of combining radiomics and dosiomics features to improve the prediction of PTP after thoracic SBRT. Clinical factors and dosimetric features did not further improve the prediction in this study. Additional immunotherapy with ICIs did not impact the prediction of PTP after thoracic SBRT.

These results could contribute to the prevention of pneumonitis by improvement of clinical decision making prior to thoracic SBRT with and without immunotherapy with ICIs.

## Data availability statement

The original contributions presented in the study are included in the article/**Supplementary Material**. Further inquiries can be directed to the corresponding author.

## Ethics statement

The studies involving human participants were reviewed and approved by 466/16S. Written informed consent for participation was not required for this study in accordance with the national legislation and the institutional requirements.

## Author contributions

KK and JP designed the project. KK and JP wrote the paper. KK and MO collected and analysed the data. JP, DB, KK and SC provided expert clinical knowledge. All authors contributed to the article and approved the submitted version.
