# Supplementary material for: Dosiomics and radiomics to predict pneumonitis after thoracic stereotactic body radiotherapy and immune checkpoint inhibition
Source: Front Oncol. 2023 Mar 15;13:1124592. doi: 10.3389/fonc.2023.1124592 (PMC10050584; doi:10.3389/fonc.2023.1124592)
Supplement: Supplementary file 1 [file Table_1.docx]

Supplementary Material

Dosiomics and Radiomics to predict Pneumonitis after Thoracic Stereotactic Body Radiotherapy and Immune Checkpoint Inhibition

Kim Melanie Kraus^1,2,3^, Maksym Oreshko^1,4^, Denise Bernhardt^1,3^, Stephanie Elisabeth Combs ^1,2,3^, Jan Caspar Peeken^1,2,3^

^1^Department of Radiation Oncology, School of Medicine and Klinikum rechts der Isar, Technical University of Munich (TUM), 81675 Munich, Germany

^2^Institute of Radiation Medicine (IRM), Helmholtz Zentrum München (HMGU) GmbH German Research Center for Environmental Health, 85764 Neuherberg, Germany

^3^Partner Site Munich, German Consortium for Translational Cancer Research (DKTK), 80336 Munich, Germany

^4^Medical Faculty, University hospital, LMU Munich, 80539 Munich, Germany

*** Correspondence:**Kim Melanie Kraus
[kimmelanie.kraus@mri.tum.de](mailto:kimmelanie.kraus@mri.tum.de)

**Supplementary Table 1: All features extracted from dose distributions or CT image data for each segment (PTV+2cm, total lung-GTV, ipsilateral lung - GTV)**

| 1 | original_shape_Elongation |
| --- | --- |
| 2 | original_shape_Flatness |
| 3 | original_shape_LeastAxisLength |
| 4 | original_shape_MajorAxisLength |
| 5 | original_shape_Maximum2DDiameterColumn |
| 6 | original_shape_Maximum2DDiameterRow |
| 7 | original_shape_Maximum2DDiameterSlice |
| 8 | original_shape_Maximum3DDiameter |
| 9 | original_shape_MeshVolume |
| 10 | original_shape_MinorAxisLength |
| 11 | original_shape_Sphericity |
| 12 | original_shape_SurfaceArea |
| 13 | original_shape_SurfaceVolumeRatio |
| 14 | original_shape_VoxelVolume |
| 15 | original_firstorder_Energy |
| 16 | original_firstorder_Entropy |
| 17 | original_firstorder_Minimum |
| 18 | original_firstorder_10Percentile |
| 19 | original_firstorder_90Percentile |
| 20 | original_firstorder_Maximum |
| 21 | original_firstorder_Mean |
| 22 | original_firstorder_Median |
| 23 | original_firstorder_InterquartileRange |
| 24 | original_firstorder_Range |
| 25 | original_firstorder_MeanAbsoluteDeviation |
| 26 | original_firstorder_RootMeanSquared |
| 27 | original_firstorder_Skewness |
| 28 | original_firstorder_Kurtosis |
| 29 | original_firstorder_Variance |
| 30 | original_firstorder_Uniformity |
| 31 | original_glcm_Autocorrelation |
| 32 | original_glcm_JointAverage |
| 33 | original_glcm_ClusterProminence |
| 34 | original_glcm_ClusterShade |
| 35 | original_glcm_ClusterTendency |
| 36 | original_glcm_Contrast |
| 37 | original_glcm_Correlation |
| 38 | original_glcm_DifferenceAverage |
| 39 | original_glcm_DifferenceEntropy |
| 40 | original_glcm_DifferenceVariance |
| 41 | original_glcm_JointEnergy |
| 42 | original_glcm_JointEntropy |
| 43 | original_glcm_Imc1 |
| 44 | original_glcm_Imc2 |
| 45 | original_glcm_Idm |
| 46 | original_glcm_Idmn |
| 47 | original_glcm_MCC |
| 48 | original_glcm_Id |
| 49 | original_glcm_Idn |
| 50 | original_glcm_InverseVariance |
| 51 | original_glcm_MaximumProbability |
| 52 | original_glcm_SumEntropy |
| 53 | original_glcm_SumSquares |
| 54 | original_glrlm_GrayLevelNonUniformity |
| 55 | original_glrlm_GrayLevelNonUniformityNormalized |
| 56 | original_glrlm_GrayLevelVariance |
| 57 | original_glrlm_HighGrayLevelRunEmphasis |
| 58 | original_glrlm_LongRunEmphasis |
| 59 | original_glrlm_LongRunHighGrayLevelEmphasis |
| 60 | original_glrlm_LongRunLowGrayLevelEmphasis |
| 61 | original_glrlm_LowGrayLevelRunEmphasis |
| 62 | original_glrlm_RunEntropy |
| 63 | original_glrlm_RunLengthNonUniformity |
| 64 | original_glrlm_RunLengthNonUniformityNormalized |
| 65 | original_glrlm_RunPercentage |
| 66 | original_glrlm_RunVariance |
| 67 | original_glrlm_ShortRunEmphasis |
| 68 | original_glrlm_ShortRunHighGrayLevelEmphasis |
| 69 | original_glrlm_ShortRunLowGrayLevelEmphasis |
| 70 | original_glszm_GrayLevelNonUniformity |
| 71 | original_glszm_GrayLevelNonUniformityNormalized |
| 72 | original_glszm_GrayLevelVariance |
| 73 | original_glszm_HighGrayLevelZoneEmphasis |
| 74 | original_glszm_LargeAreaEmphasis |
| 75 | original_glszm_LargeAreaHighGrayLevelEmphasis |
| 76 | original_glszm_LargeAreaLowGrayLevelEmphasis |
| 77 | original_glszm_LowGrayLevelZoneEmphasis |
| 78 | original_glszm_SizeZoneNonUniformity |
| 79 | original_glszm_SizeZoneNonUniformityNormalized |
| 80 | original_glszm_SmallAreaEmphasis |
| 81 | original_glszm_SmallAreaHighGrayLevelEmphasis |
| 82 | original_glszm_SmallAreaLowGrayLevelEmphasis |
| 83 | original_glszm_ZoneEntropy |
| 84 | original_glszm_ZonePercentage |
| 85 | original_glszm_ZoneVariance |
| 86 | original_ngtdm_Busyness |
| 87 | original_ngtdm_Coarseness |
| 88 | original_ngtdm_Complexity |
| 89 | original_ngtdm_Contrast |
| 90 | original_ngtdm_Strength |
| 91 | original_gldm_DependenceEntropy |
| 92 | original_gldm_DependenceNonUniformity |
| 93 | original_gldm_DependenceNonUniformityNormalized |
| 94 | original_gldm_DependenceVariance |
| 95 | original_gldm_GrayLevelNonUniformity |
| 96 | original_gldm_GrayLevelVariance |
| 97 | original_gldm_HighGrayLevelEmphasis |
| 98 | original_gldm_LargeDependenceEmphasis |
| 99 | original_gldm_LargeDependenceHighGrayLevelEmphasis |
| 100 | original_gldm_LargeDependenceLowGrayLevelEmphasis |
| 101 | original_gldm_LowGrayLevelEmphasis |
| 102 | original_gldm_SmallDependenceEmphasis |
| 103 | original_gldm_SmallDependenceHighGrayLevelEmphasis |
| 104 | original_gldm_SmallDependenceLowGrayLevelEmphasis |

# Supplementary Table 2: all extracted features after feature reduction used in this study. Abbreviations used: planning target volume (PTV), total lung-GTV (TL), ipsilateral lung-GTV (IL)

| Features | Physical dose | EQD2 |
| --- | --- | --- |
| Clinical Features | Size  Age  Location  Sex | Size  Age  Location  Sex |
| Dosimetric Features | V50 | V5 |
| Dosimetric Features & Clinical Features | V50  Size  Age  Location  Sex | Size  Age  Location  Sex  V5 |
| Dosiomics Features | PTV_original_shape_Sphericity  TL_original_shape_Flatness  TL_original_shape_Elongation  IL_original_shape_Flatness  PTV_original_glszm_SmallAreaLowGrayLevelEmphasis  PTV_original_glcm_InverseVariance  TL_original_glcm_Imc1  PTV_original_shape_Flatness  PTV_original_glszm_LowGrayLevelZoneEmphasis  IL_original_shape_Maximum2DDiameterSlice  IL_original_shape_Elongation  IL_original_glrlm_ShortRunLowGrayLevelEmphasis  PTV_original_ngtdm_Contrast  IL_original_glrlm_LongRunLowGrayLevelEmphasis  PTV_original_glszm_ZoneEntropy  IL_original_glrlm_RunEntropy  TL_original_glszm_LargeAreaLowGrayLevelEmphasis | PTV_original_shape_Sphericity  TL_original_shape_Flatness  PTV_original_glcm_Idmn  TL_original_shape_Elongation  TL_original_glcm_Imc1  IL_original_shape_Flatness  IL_original_glrlm_RunEntropy  IL_original_shape_Elongation  PTV_original_gldm_DependenceVariance  PTV_original_shape_Flatness  IL_original_shape_Maximum2DDiameterSlice  PTV_original_firstorder_Minimum  PTV_original_ngtdm_Contrast  PTV_original_gldm_DependenceNonUniformityNormalized  TL_original_glszm_LargeAreaHighGrayLevelEmphasis  IL_original_glrlm_ShortRunLowGrayLevelEmphasis  TL_original_glszm_SmallAreaEmphasis |
| Radiomics Features | PTV_original_shape_Sphericity  TL_original_glcm_Idn  IL_original_glcm_InverseVariance  TL_original_shape_Elongation  TL_original_shape_Flatness  PTV_original_firstorder_Kurtosis  IL_original_shape_Flatness  PTV_original_glcm_Correlation  PTV_original_shape_Flatness  PTV_original_glszm_SmallAreaEmphasis  IL_original_ngtdm_Contrast  PTV_original_glcm_ClusterProminence  PTV_original_ngtdm_Contrast  IL_original_shape_Elongation  PTV_original_glszm_GrayLevelVariance  PTV_original_firstorder_InterquartileRange  TL_original_glszm_ZonePercentage  IL_original_firstorder_Minimum  PTV_original_glcm_MCC  PTV_original_glcm_DifferenceVariance  TL_original_glcm_Imc1  TL_original_shape_Maximum2DDiameterSlice | PTV_original_shape_Sphericity  TL_original_glcm_Idn  IL_original_glcm_InverseVariance  TL_original_shape_Elongation  TL_original_shape_Flatness  IL_original_shape_Flatness  PTV_original_firstorder_Kurtosis  PTV_original_glcm_Correlation  PTV_original_shape_Flatness  PTV_original_glszm_SmallAreaEmphasis  IL_original_ngtdm_Contrast  PTV_original_glcm_ClusterProminence  PTV_original_ngtdm_Contrast  IL_original_shape_Elongation  PTV_original_glszm_GrayLevelVariance  IL_original_firstorder_Minimum  TL_original_glszm_ZonePercentage  PTV_original_glcm_MCC  PTV_original_firstorder_InterquartileRange  TL_original_glcm_Imc1  PTV_original_glcm_DifferenceVariance  PTV_original_gldm_LargeDependenceHighGrayLevelEmphasis |
| Dosiomics+Radiomics Features | PTV_original_shape_Sphericity  PTV_original_glszm_SmallAreaLowGrayLevelEmphasis  IL_original_glcm_InverseVariance  TL_original_glcm_Idn  PTV_original_glcm_Correlation  PTV_original_glszm_LowGrayLevelZoneEmphasis  TL_original_shape_Flatness  PTV_original_firstorder_Kurtosis  PTV_original_glcm_InverseVariance  PTV_original_shape_Sphericity  PTV_original_ngtdm_Contrast  TL_original_glcm_Imc1  IL_original_ngtdm_Contrast  TL_original_shape_Elongation  TL_original_glszm_ZonePercentage  PTV_original_glcm_ClusterProminence  PTV_original_glszm_SmallAreaEmphasis  PTV_original_glcm_MCC  PTV_original_firstorder_InterquartileRange  IL_original_glrlm_ShortRunLowGrayLevelEmphasis  TL_original_glcm_Imc1  PTV_original_glszm_GrayLevelVariance  PTV_original_gldm_LargeDependenceHighGrayLevelEmphasis  IL_original_glrlm_LongRunLowGrayLevelEmphasis  TL_original_shape_Flatness  PTV_original_ngtdm_Contrast  IL_original_glszm_LargeAreaHighGrayLevelEmphasis  IL_original_shape_Flatness | PTV_original_shape_Sphericity  PTV_original_glcm_Idmn  IL_original_glcm_InverseVariance  TL_original_glcm_Idn  TL_original_glcm_Imc1  TL_original_shape_Elongation  PTV_original_glcm_Correlation  PTV_original_firstorder_Kurtosis  TL_original_shape_Flatness  PTV_original_ngtdm_Contrast  IL_original_glrlm_RunEntropy  PTV_original_glszm_SmallAreaEmphasis  PTV_original_gldm_DependenceNonUniformityNormalized  TL_original_glszm_LargeAreaHighGrayLevelEmphasis  IL_original_ngtdm_Contrast  PTV_original_glcm_ClusterProminence  TL_original_glszm_ZonePercentage  PTV_original_glcm_MCC  IL_original_shape_Flatness  PTV_original_firstorder_InterquartileRange  IL_original_shape_Elongation  IL_original_glrlm_ShortRunLowGrayLevelEmphasis  PTV_original_ngtdm_Contrast  PTV_original_gldm_DependenceVariance  TL_original_glcm_Imc1  PTV_original_glszm_GrayLevelVariance  PTV_original_gldm_LargeDependenceHighGrayLevelEmphasis  TL_original_shape_Flatness |
| Radiomics+DVH+Clinical Features | PTV_original_shape_Sphericity  TL_original_glcm_Idn  IL_original_glcm_InverseVariance  TL_original_shape_Elongation  TL_original_shape_Flatness  PTV_original_firstorder_Kurtosis  IL_original_shape_Flatness  PTV_original_glcm_Correlation  PTV_original_shape_Flatness  PTV_original_glszm_SmallAreaEmphasis  IL_original_ngtdm_Contrast  PTV_original_glcm_ClusterProminence  PTV_original_ngtdm_Contrast  IL_original_shape_Elongation  PTV_original_glszm_GrayLevelVariance  PTV_original_firstorder_InterquartileRange  TL_original_glszm_ZonePercentage  IL_original_firstorder_Minimum  PTV_original_glcm_MCC  PTV_original_glcm_DifferenceVariance  TL_original_glcm_Imc1  TL_original_shape_Maximum2DDiameterSlice  V50  Size  Age  Location  Sex | PTV_original_shape_Sphericity  TL_original_glcm_Idn  IL_original_glcm_InverseVariance  TL_original_shape_Elongation  TL_original_shape_Flatness  IL_original_shape_Flatness  PTV_original_firstorder_Kurtosis  PTV_original_glcm_Correlation  PTV_original_shape_Flatness  PTV_original_glszm_SmallAreaEmphasis  IL_original_ngtdm_Contrast  PTV_original_glcm_ClusterProminence  PTV_original_ngtdm_Contrast  IL_original_shape_Elongation  PTV_original_glszm_GrayLevelVariance  IL_original_firstorder_Minimum  TL_original_glszm_ZonePercentage  PTV_original_glcm_MCC  PTV_original_firstorder_InterquartileRange  TL_original_glcm_Imc1  PTV_original_glcm_DifferenceVariance  PTV_original_gldm_LargeDependenceHighGrayLevelEmphasis  V5  Size  Age  Location  Sex |
| Dosiomics+DVH+ClinicalFactors | PTV_original_shape_Sphericity  TL_original_shape_Flatness  TL_original_shape_Elongation  IL_original_shape_Flatness  PTV_original_glszm_SmallAreaLowGrayLevelEmphasis  PTV_original_glcm_InverseVariance  TL_original_glcm_Imc1  PTV_original_shape_Flatness  PTV_original_glszm_LowGrayLevelZoneEmphasis  IL_original_shape_Maximum2DDiameterSlice  IL_original_shape_Elongation  IL_original_glrlm_ShortRunLowGrayLevelEmphasis  PTV_original_ngtdm_Contrast  IL_original_glrlm_LongRunLowGrayLevelEmphasis  PTV_original_glszm_ZoneEntropy  IL_original_glrlm_RunEntropy  TL_original_glszm_LargeAreaLowGrayLevelEmphasis  V50  Size  Age  Location  Sex | PTV_original_shape_Sphericity  TL_original_shape_Flatness  TL_original_shape_Elongation  PTV_original_glcm_Idmn  TL_original_glcm_Imc1  IL_original_shape_Flatness  PTV_original_gldm_DependenceVariance  IL_original_shape_Elongation  IL_original_glrlm_RunEntropy  PTV_original_shape_Flatness  IL_original_shape_Maximum2DDiameterSlice  PTV_original_firstorder_Minimum  PTV_original_ngtdm_Contrast  PTV_original_gldm_DependenceNonUniformityNormalized  TL_original_glszm_LargeAreaHighGrayLevelEmphasis  IL_original_glrlm_ShortRunLowGrayLevelEmphasis  TL_original_glszm_SmallAreaEmphasis  V5  Size  Alter  Location  Sex |
| Dosiomics+Radiomics+DVH+Clinical Features | PTV_original_shape_Sphericity  PTV_original_glszm_SmallAreaLowGrayLevelEmphasis  IL_original_glcm_InverseVariance  TL_original_glcm_Idn  PTV_original_glcm_Correlation  PTV_original_glszm_LowGrayLevelZoneEmphasis  TL_original_shape_Flatness  PTV_original_firstorder_Kurtosis  PTV_original_glcm_InverseVariance  PTV_original_shape_Sphericity  PTV_original_ngtdm_Contrast  TL_original_glcm_Imc1  IL_original_ngtdm_Contrast  TL_original_shape_Elongation  TL_original_glszm_ZonePercentage  PTV_original_glcm_ClusterProminence  PTV_original_glszm_SmallAreaEmphasis  PTV_original_glcm_MCC  PTV_original_firstorder_InterquartileRange  IL_original_glrlm_ShortRunLowGrayLevelEmphasis  TL_original_glcm_Imc1  PTV_original_glszm_GrayLevelVariance  PTV_original_gldm_LargeDependenceHighGrayLevelEmphasis  IL_original_glrlm_LongRunLowGrayLevelEmphasis  TL_original_shape_Flatness  PTV_original_ngtdm_Contrast  IL_original_glszm_LargeAreaHighGrayLevelEmphasis  IL_original_shape_Flatness  V50  Size  Age  Location  Sex | PTV_original_shape_Sphericity  PTV_original_glcm_Idmn  IL_original_glcm_InverseVariance  TL_original_glcm_Idn  TL_original_glcm_Imc1  TL_original_shape_Elongation  PTV_original_glcm_Correlation  PTV_original_firstorder_Kurtosis  TL_original_shape_Flatness  PTV_original_ngtdm_Contrast  IL_original_glrlm_RunEntropy  PTV_original_glszm_SmallAreaEmphasis  PTV_original_gldm_DependenceNonUniformityNormalized  TL_original_glszm_LargeAreaHighGrayLevelEmphasis  IL_original_ngtdm_Contrast  PTV_original_glcm_ClusterProminence  TL_original_glszm_ZonePercentage  PTV_original_glcm_MCC  IL_original_shape_Flatness  PTV_original_firstorder_InterquartileRange  IL_original_shape_Elongation  IL_original_glrlm_ShortRunLowGrayLevelEmphasis  PTV_original_ngtdm_Contrast  PTV_original_gldm_DependenceVariance  TL_original_glcm_Imc1  PTV_original_glszm_GrayLevelVariance  PTV_original_gldm_LargeDependenceHighGrayLevelEmphasis  TL_original_shape_Flatness  V5  Size  Age  Location  Sex |

#

# Supplementary Table 3: Area under the receiver operating curve (AUC) values for physical dose and equivalent uniform dose (EQD2) without Synthetic Minority Oversampling Technique (SMOTE).

| **Dose P** | **Dose Parameter** | **Physical dose** | | **EQD2** | |
| --- | --- | --- | --- | --- | --- |
|  | **Classifier:** | **rf** | **glmnet** | **rf** | **glmnet** |
|  | **Model** |  |  |  |  |
|  | Radiomics | 0.74 |  | 0.74 |  |
|  | Dosiomics | 0.69 |  | 0.68 |  |
|  | Clinical factors | 0.42 |  | 0.42 |  |
|  | DVH |  | 0.40 |  | 0.40 |
|  | ICI |  | 0.47 |  | 0.47 |
|  | Dosiomics +Radiomics | 0.79 |  | 0.78 |  |
|  | DVH+Clinical factors | 0.44 |  | 0.47 |  |
|  | Radiomics+DVH+Clinical factors | 0.72 |  | 0.73 |  |
|  | Dosiomics+DVH+Clinical factors | 0.66 |  | 0.66 |  |
|  | All | 0.78 |  | 0.77 |  |

# Supplementary Table 4: Hyperparemeter tuning grid for machine learning models

|  | **Hyperparameter** | **Search space  (by increment)** |
| --- | --- | --- |
| **Elastic Net Regression** | Lambda | 0 – 1, by 0.02 |
|  | Alpha | 0 – 1, by 0.05 |
| **Random Forest** | Number of trees (ntree) | 501 – 2001, by 500 |
|  | Features per node (Mtry) | 1 – 6, by 1 |
| **LogitBoost** | Iteration Number | 1 – 100, by 2 |
| **Support Vector Machine** | C | 0.00001 – 0.01. by 0.005, 0.01 – 0.1, by 0.05,  0.1 – 1, by 0.2,  1 – 10, by 2 |
|  | Sigma | 0.1 – 1, by 0.1,  1 –50, by 5,  60 – 450, by 70 |

# Supplementary Table 4: Spearman correlation coefficients between additional immune checkpoint inhibition (ICI) and all predictive features within the model combining all features classes

| PTV_original_shape_Sphericity | -0.20088938158622 |
| --- | --- |
| PTV_original_glszm_SmallAreaLowGrayLevelEmphasis | -0.167487346643143 |
| IL_original_glcm_InverseVariance | 0.110226466844211 |
| GL_original_glcm_Idn | 0.046285572657526 |
| PTV_original_glcm_Correlation | -0.187528567608989 |
| PTV_original_glszm_LowGrayLevelZoneEmphasis | -0.0835050873576925 |
| GL_original_shape_Flatness | -0.0033401959649761 |
| PTV_original_firstorder_Kurtosis | -0.0214727367491209 |
| PTV_original_glcm_InverseVariance | -0.0615551786808133 |
| PTV_original_shape_Sphericity | -0.194208974597605 |
| PTV_original_ngtdm_Contrast | 0.253378293639627 |
| TL_original_glcm_Imc1 | -0.147445793311088 |
| IL_original_ngtdm_Contrast | -0.0558289897003149 |
| TL_original_shape_Elongation | 0.117861200478442 |
| GL_original_glszm_ZonePercentage | -0.108317783435654 |
| PTV_original_glcm_ClusterProminence | -0.0405596138594506 |
| PTV_original_glszm_SmallAreaEmphasis | 0.0119292981939561 |
| PTV_original_glcm_MCC | -0.137902687122132 |
| PTV_original_firstorder_InterquartileRange | -0.0066804822834589 |
| IL_original_glrlm_ShortRunLowGrayLevelEmphasis | 0.0472399143618049 |
| GL_original_glcm_Imc1 | 0.0338791305019005 |
| PTV_original_glszm_GrayLevelVariance | 0.0405596138594506 |
| PTV_original_gldm_LargeDependenceHighGrayLevelEmphasis | 0.0348335507263517 |
| IL_original_glrlm_LongRunLowGrayLevelEmphasis | 0.0987743663928647 |
| TL_original_shape_Flatness | -0.0033401959649761 |
| PTV_original_ngtdm_Contrast | -0.129313592422484 |
| IL_original_glszm_LargeAreaHighGrayLevelEmphasis | 0.0892309493500759 |
| IL_original_shape_Flatness | -0.199934587046427 |
| V50 | -0.00858913342231794 |
| Size | 0.0968659013349233 |
| Age | -0.0601798503316456 |
| Location | 0.155169375157095 |
| Sex | 0.0564033346637209 |
